# Left Atrial Size Modification After Catheter Ablation Predicts Late Atrial Fibrillation Recurrence

**DOI:** 10.3390/diagnostics16040628

**Published:** 2026-02-21

**Authors:** Yung-Lung Chen, Bang-Yan Hou, Hsiang-Hsuan Chen, Pei-Ting Lin, Hui-Ting Wang

**Affiliations:** 1 Section of Cardiology, Department of Internal Medicine, Kaohsiung Chang Gung Memorial Hospital, Ksohsiung 833401, Taiwan; feymanchen@gmail.com (Y.-L.C.); k03149@cgmh.org.tw (B.-Y.H.); samuel13531@cgmh.org.tw (H.-H.C.); r40391132@gmail.com (P.-T.L.); 2 Graduate Institute of Clinical Medical Sciences, College of Medicine, Chang Gung University, Taoyuan 333323, Taiwan; 3 School of Medicine, College of Medicine, National Sun Yat-sen University, Kaohsiung 804201, Taiwan; 4 Emergency Department, Kaohsiung Chang Gung Memorial Hospital, Ksohsiung 833401, Taiwan

**Keywords:** atrial fibrillation, radiofrequency ablation, left atrial volume index, remodeling, late recurrence

## Abstract

**Background**: Radiofrequency catheter ablation for atrial fibrillation (AF) restores sinus rhythm, but late recurrence is common. Left atrial (LA) size is a known predictor of AF recurrence, but the prognostic value of early post-ablation LA remodeling remains underexplored. **Objective**: We aimed to evaluate whether pre-ablation and early post-ablation LA volume index (LAVI) predict late atrial tachyarrhythmia recurrence after AF ablation. **Methods**: This is a retrospective single-center study of adults undergoing their first radiofrequency ablation for AF between January 2013 and December 2021. LA volume was measured by transthoracic echocardiography and indexed to body surface area to derive LAVI within one week before ablation and at 6 and 12 months after the procedure. The 6-month echocardiographic assessment was prespecified as the primary early post-ablation time point because it occurs beyond the 3-month blanking period and captures early structural remodeling during routine follow-up. Early recurrence was defined as atrial tachyarrhythmia occurring within 3 months after ablation, and late recurrence (LR) as any atrial tachyarrhythmic event thereafter. Multivariable Cox proportional hazards models were used to identify independent predictors of LR. **Results**: Among 408 patients with at least one year of follow-up, 157 (38.5%) experienced LR. Age and sex were similar between recurrence and non-recurrence groups (60.7 ± 9.8 vs. 59.9 ± 0.8 years; 56.1% vs. 64.1% male). Recurrence was associated with a higher prevalence of atrial flutter and persistent AF, higher pre-ablation and post-ablation LAVI, and lower post-ablation left ventricular ejection fraction. In multivariable analysis, atrial flutter, persistent AF, and LAVI, measured both before and after ablation, were independent predictors of LR. In receiver operating characteristic analysis, pre-ablation LAVI demonstrated modest discrimination for LR (AUC = 0.622; 95% CI 0.563–0.681; *p* < 0.001), with an optimal cut-off of 41.6 mL/m^2^, while post-ablation LAVI showed similar performance (AUC = 0.597; 95% CI 0.532–0.662; *p* = 0.003), with a cut-off of 38.6 mL/m^2^. Overall, discrimination was modest (AUC < 0.65), limiting LAVI as a standalone predictor. **Conclusions**: Elevated LAVI measured before and early after AF ablation independently predicts LR. Limited post-ablation LA reverse remodeling, reflected by persistently increased LAVI, is associated with unfavorable long-term rhythm outcomes. Serial assessment of LAVI may enhance post-ablation risk stratification.

## 1. Introduction

Atrial fibrillation (AF) is one of the most common cardiac arrhythmias encountered clinically, imposing a significant burden on public health and leading to severe complications such as thromboembolism and heart failure (HF). Specifically, AF is associated with approximately a five-fold increase in the risk of ischemic stroke [1]. With the progression of AF, the left atrium (LA) gradually enlarges and undergoes functional deterioration, further elevating the risk of HF. Radiofrequency catheter ablation (RFA) has been established as an effective treatment strategy to maintain sinus rhythm in AF patients, improving cardiac function and quality of life, and demonstrating superior efficacy compared to antiarrhythmic medications [2]. Nevertheless, despite its widespread adoption, RFA remains associated with considerable recurrence rates, particularly among patients with persistent AF [3,4]. These recurrence events diminish the long-term effectiveness of ablation therapy and often necessitate repeat procedures, underscoring the critical need for reliable predictors of AF recurrence.

Previous studies have consistently shown that a larger baseline LA size is significantly associated with an increased risk of arrhythmia recurrence following AF ablation [5]. A meta-analysis demonstrated that patients with recurrence had substantially larger average LA volumes than those without recurrence, with greater LA volumes correlating with higher postoperative AF recurrence rates [5]. However, these identified predictors are predominantly static preprocedural structural parameters. Theoretically, successful ablation and sustained sinus rhythm could induce structural “reverse remodeling,” characterized by reductions in LA size and improvements in function. However, whether postoperative LA structural changes can reliably predict AF recurrence remains inconclusive, as this question has been addressed by only a limited number of studies to date [6]. Demonstrating the prognostic value of monitoring postoperative LA remodeling could facilitate early identification of high-risk patients, allowing timely adjustments to therapeutic strategies and thereby enhancing postoperative management outcomes.

Against this background, our single-center retrospective study aimed to investigate whether changes in LA size following RFA predict AF recurrence. We compare preprocedural and follow-up LA size measurements and analyze their association with AF recurrence to provide insights that inform risk stratification and guide clinical decision-making. In contrast to prior work focused mainly on baseline LA size, we specifically evaluated the prognostic value of early post-ablation structural reverse remodeling using serial echocardiographic LAVI. We hypothesized that reductions in LA size after ablation (i.e., reverse remodeling) would be associated with a lower risk of AF recurrence.

## 2. Materials and Methods

### 2.1. Study Design and Population

This retrospective, single-center cohort study was conducted at Kaohsiung Chang Gung Memorial Hospital. The study included consecutive adult patients (aged ≥ 18 years) with a documented diagnosis of AF who underwent their first RFA for rhythm control between January 2013 and December 2021. Persistent AF was defined as AF lasting ≥ 7 days or requiring pharmacologic or electrical cardioversion. Patients were excluded if they had undergone previous AF ablation, had significant structural heart disease, or lacked echocardiographic data at baseline or follow-up.

Transthoracic echocardiography was performed by experienced echocardiographers blinded to clinical outcomes within one week before ablation, and repeated at 6 and 12 months after the procedure. Left atrial (LA) size was assessed using the left atrial volume index (LAVI). LA volume was measured at ventricular end-systole (just before mitral valve opening) using the biplane method of disks (modified Simpson’s rule) from apical four- and two-chamber views, in accordance with contemporary echocardiographic chamber quantification recommendations. The LA endocardial border was traced with exclusion of the pulmonary veins and the left atrial appendage. LAVI was calculated as LA volume divided by body surface area (BSA) and expressed as mL/m^2^ [7]. Only patients with complete pre- and post-procedural LA measurements and a minimum of 12 months of clinical follow-up were included in the analysis. The Institutional Review Board of Chang Gung Medical Foundation approved the study protocol and granted a waiver of additional consent for retrospective data analysis (IRB number: 202501260B0). The study protocol adhered to the ethical principles outlined in the Declaration of Helsinki, as revised in 2024, and was approved by the local ethics committee. All patient details were fully de-identified to ensure anonymity and prevent any potential identification.

### 2.2. Radiofrequency Ablation Procedures

All ablation procedures were conducted under conscious sedation and local anesthesia. Vascular access was established via the femoral vein, followed by transseptal puncture under intracardiac echocardiographic or fluoroscopic guidance to access the LA. A three-dimensional electroanatomic map of the LA was constructed using the EnSite Precision system (Abbott, Chicago, IL, USA). Wide-area circumferential ablation lesions were deployed around the ipsilateral pulmonary vein (PV) antra to achieve PV isolation (PVI). In cases where AF persisted after PVI, electrical cardioversion was performed to restore a sinus rhythm. Subsequently, isoproterenol infusion was administered to provoke non-PV triggers. Any inducible focal atrial tachycardia (AT) or ectopic firing site, including those originating from the superior vena cava or other atrial regions, was mapped and ablated. Macroreentrant AT or atrial flutter (AFL), whether documented before or induced during the procedure, was targeted using activation mapping, with ablation performed as appropriate. For patients presenting with persistent AF or exhibiting immediate recurrence of AF following cardioversion without identifiable triggers, adjunctive substrate modification was undertaken. This stepwise approach included empirical isolation of the posterior LA wall, ablation of complex fractionated atrial electrograms, and ethanol infusion into the vein of Marshall. These interventions were sequentially applied until AF was rendered non-inducible. All procedures were performed by experienced electrophysiologists in accordance with contemporary clinical guidelines.

Contemporary three-dimensional mapping systems and intracardiac echocardiography can facilitate minimal- or zero-fluoroscopy AF ablation workflows. Although electroanatomic mapping platforms may also provide an estimate of intracavitary LA volume from the reconstructed geometry, mapping-based volumes depend on point density, rhythm at acquisition, and segmentation (e.g., how pulmonary veins/appendage are excluded) and are not directly interchangeable with echocardiography-derived LA volumes for longitudinal assessment; validation studies have shown systematic biases between invasive (angiography/electroanatomic mapping) and echocardiographic LA volume measurements [8]. Accordingly, LA size in this study was assessed exclusively by standardized transthoracic echocardiography (LAVI) at baseline and during follow-up.

### 2.3. Follow-Up and Outcome Definitions

All patients underwent longitudinal follow-up for at least 12 months after catheter ablation. Standard 12-lead electrocardiograms (ECGs) were obtained at 1 week, 1 month, and subsequently at 3-month intervals. Additionally, 24-h Holter monitoring was performed between 3 and 6 months after catheter ablation, and, in cases where patients reported arrhythmic symptoms, at the treating physician’s discretion. Transthoracic echocardiography was repeated at 6 and 12 months to evaluate changes in LAVI and left ventricular ejection fraction (LVEF). Early recurrence (ER) was defined as any documented atrial tachyarrhythmia—including AF, AFL, or AT lasting ≥ 30 s and occurring within the initial 3-month post-procedural blanking period. Late recurrence (LR) was defined as any atrial tachyarrhythmia of similar duration arising beyond this 3-month window. The primary endpoint was the incidence of LR during follow-up. Patients who experienced ER were not classified as procedural failures and were monitored beyond the blanking period for subsequent arrhythmic events. Individuals with ER who remained free of atrial tachyarrhythmia for at least 3 months post-ablation were adjudicated as having no primary outcome event.

### 2.4. Statistical Analysis

Continuous variables were summarized as mean ± standard deviation and compared between patients with and without LR using the independent-samples Student’s *t*-test. Categorical variables were presented as counts (percentages) and compared with the χ^2^ test or Fisher’s exact test, as appropriate. To evaluate the discriminative ability of pre- and post-ablation LAVI for predicting LR, receiver operating characteristic (ROC) curves were generated, and the area under the curve (AUC) with 95% confidence intervals (CI) was calculated. Optimal cut-off values were determined using the Youden index. Variables with *p* < 0.10 on univariable analysis to predict LR were entered into multivariable Cox proportional hazards models. Two separate models were constructed to avoid multicollinearity between pre- and post-ablation LAVI: Model 1 included baseline covariates plus pre-ablation LAVI, while Model 2 included the same covariates with post-ablation LAVI measured at the 6-month follow-up, instead of the pre-ablation value. LAVI changes over time were analyzed using paired *t*-tests and repeated-measures ANOVA, while differences between recurrence and non-recurrence groups were assessed with independent *t*-tests. The proportional hazards assumption was assessed using Schoenfeld residuals. Stepwise variable selection guided by the Akaike Information Criterion (AIC) was applied for model optimization, and multicollinearity was assessed using variance inflation factors (VIF). Statistical analyses were performed using SPSS version 25.0 (IBM Corp., Armonk, NY, USA) and R (R Foundation for Statistical Computing, Vienna, Austria).

## 3. Results

### 3.1. Study Population and Baseline Characteristics

From January 2013 to December 2021, 408 patients undergoing catheter ablation for AF were included after excluding 13 patients who were lost to follow-up. The average follow-up duration was 31.7 ± 23.8 months. LR of AF occurred in 157 patients, whereas 251 remained free of LR during at least 1 year of follow-up. Overall, LR occurred in 38.5% of the cohort during long-term follow-up. Baseline characteristics are summarized in Table 1. There was no significant difference in age and sex distribution between patients with and without late recurrence. The prevalence of hypertension, diabetes mellitus, HF, prior stroke, and coronary artery disease did not differ between groups (all *p* > 0.05). In contrast, a history of AFL (36.9% vs. 21.5%; *p* < 0.001) and persistent AF (38.9% vs. 19.5%; *p* < 0.001) was more frequent in the LR group. The CHA_2_DS_2_ VASc score was slightly higher in patients with LR (2.2 ± 1.5 vs. 1.8 ± 1.5; *p* = 0.019). Echocardiography showed significantly larger LAVI in the LR group both before ablation (46.1 ± 22.3 vs. 36.9 ± 15.6 mL/m^2^; *p* < 0.001) and 6 months after ablation (44.6 ± 25.6 vs. 36.2 ± 17.5 mL/m^2^; *p* < 0.001). LVEF before ablation did not differ (64.8 ± 12.0 vs. 67.8 ± 9.9%; *p* = 0.074), but was lower in the LR group at follow-up (65.6 ± 11.4 vs. 69.7 ± 8.8%; *p* = 0.008).

### 3.2. Multivariable Regression Analysis

To identify independent predictors of LR, two Cox proportional hazards models were constructed (Table 2). Model 1 included baseline characteristics and pre-ablation echocardiographic parameters with *p* < 0.10 on univariate analysis. AFL (Hazard Ratios (HRs): 1.473; 95% CI: 1.06–2.05; *p* = 0.021), persistent AF (HRs: 2.029; 95% CI: 1.42–2.90; *p* < 0.001), and pre-ablation LA size (HRs: 1.044 per mm increase; 95% CI: 1.01–1.07; *p* = 0.004) were all independently associated with LR. In Model 2, using post-ablation LA measurements in place of pre ablation values, AFL (HRs: 1.554; 95% CI: 1.09–2.21; *p* = 0.015), persistent AF (HRs: 1.797; 95% CI: 1.25–2.58; *p* < 0.001) and post-ablation LA size (HRs: 1.031 per mm increase; 95% CI: 1.00–1.06; *p* = 0.033) remained significant predictors. Stroke history, CHA_2_DS_2_ VASc ≥ 2, and LVEF (before or after ablation) were not significantly associated with LR in either model. The proportional hazards assumption was satisfied for all covariates, as confirmed by nonsignificant Schoenfeld residual tests (Supplementary Figure S1). Model 1 (using pre-ablation LA size) and Model 2 (using post-ablation LA size) both demonstrated acceptable overall fit and similar discriminatory ability. Stepwise AIC selection yielded the final variable sets, and no variable showed significant multicollinearity (all VIFs < 2.0) (Supplementary Tables S1 and S2). These results confirm that the Cox models were stable and the HR estimates were reliable.

### 3.3. Predictive Performance of LA Size

**Figure 1:**
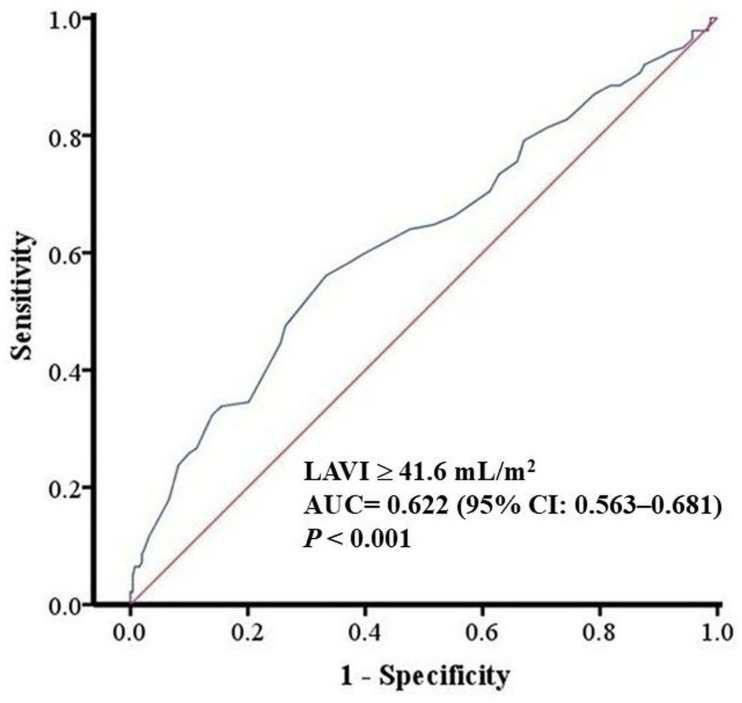
ROC curve of baseline LAVI for predicting late recurrence after AF ablation (AUC 0.622; optimal cut-off 41.6 mL/m^2^). AUC = area under the curve; CI = confidence interval; LAVI = left atrial volume index.

**Figure 2:**
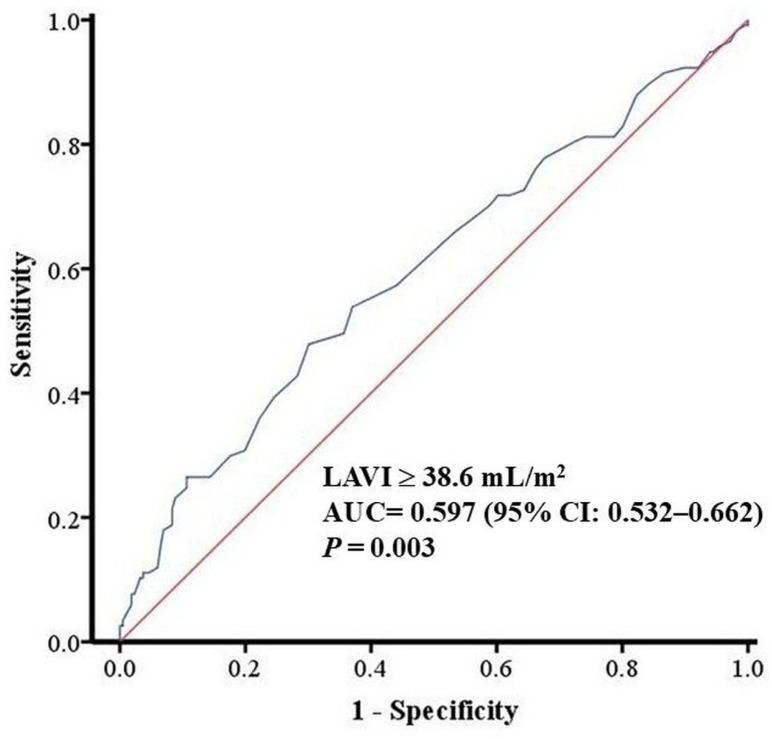
Receiver operating characteristic curve of post-ablation left atrial volume index (LAVI) for predicting late recurrence of atrial fibrillation (AUC 0.597; cut-off 38.6 mL/m^2^).

ROC curves were constructed to evaluate the discriminative ability of left atrial volume index (LAVI) for predicting LR. The AUC for pre-ablation LAVI was 0.622 (95% CI 0.563–0.681; *p* < 0.001), with an optimal cut-off of 41.6 mL/m^2^ (Figure 1). For post-ablation LAVI, the AUC was 0.597 (95% CI 0.532–0.662; *p* = 0.003), and the best cut-off was 38.6 mL/m^2^ (Figure 2).

### 3.4. LA Reverse Remodeling Within One Year After Radiofrequency Ablation in Patients With and Without LR

**Figure 3:**
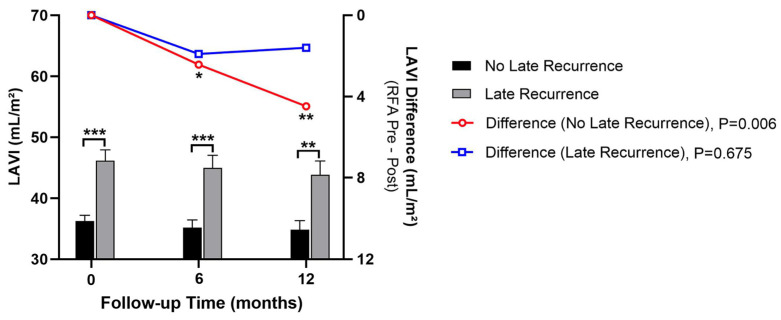
Left atrial volume index (LAVI) remodeling after ablation in patients with and without late arrhythmia recurrence. This figure shows mean LAVI (mL/m^2^) at baseline and at 6 and 12 months post-ablation in the no late recurrence group (black bars) and the late recurrence group (grey bars). Error bars indicate mean ± 1 SD. The lines depict LAVI difference (pre–post) in each group; the change over time was significant in the no late recurrence group (*p* = 0.006) but not in the late recurrence group (*p* = 0.675). Asterisks denote statistical significance (* *p* < 0.05; ** *p* < 0.01; *** *p* < 0.001) for comparisons as specified in the analysis. LAVI = left atrial volume index; LR = late recurrence.

The dynamic change in LAVI from baseline to 6 and 12 months after ablation is illustrated in Figure 3. Patients without LR demonstrated a significant change in LAVI difference (pre–post) over time (*p* = 0.006), whereas those with LR did not (*p* = 0.675), suggesting less pronounced reverse remodeling in the recurrence group. Patients without LR exhibited a significant reduction in LAVI, consistent with favorable reverse remodeling, whereas those with LR demonstrated minimal or no decrease in LAVI. This divergence highlights that the degree of LA structural reverse remodeling during the first year is closely linked to long-term rhythm outcome after ablation.

## 4. Discussion

In this single-center cohort of AF ablation patients, we found that both pre- and early post-ablation LA size (6 months post-RFA) were significant predictors of late AF recurrence (beyond the 3-month blanking period). The observed divergence in LA size trajectories between patients with and without recurrence provides visual evidence that early reverse remodeling after ablation carries prognostic value. Those who experienced a measurable reduction in LAVI within the first 6 months tended to maintain sinus rhythm during midterm follow-up (31.7 ± 23.8 months, or an average of 2.6 years), suggesting that early structural reverse remodeling reflects effective substrate modification. Moreover, sustained sinus rhythm after RFA appeared to facilitate continuous structural reverse remodeling for at least one year in our cohort, underscoring the dynamic nature of atrial recovery following successful ablation. In contrast, patients whose LA size failed to regress exhibited a higher risk of recurrence, implying ongoing atrial pathology or incomplete reverse remodeling. However, given the modest discriminative ability of LAVI alone (AUC: 0.622), it is not sufficient as a stand-alone predictor. Accordingly, LAVI should not be used as a standalone decision tool, but rather integrated with clinical phenotype (e.g., persistent AF, AFL history) and other substrate markers. Therefore, a multi-modal approach—integrating additional modalities such as fibrosis imaging, atrial strain, or electrophysiological mapping—is necessary to improve risk stratification for AF recurrence. Patients who experienced recurrence had larger LAVI on echocardiography both before ablation and at 6 and 12 months after ablation, compared to those who remained in sinus rhythm. Notably, each 1 mL/m^2^ increase in LAVI was associated with approximately a 1.4% higher risk of recurrence on multivariable analysis. This study is one of the first to evaluate LA size at both baseline and follow-up as predictors of LR, highlighting the novel insight that post-ablation LA remodeling (or lack thereof) carries prognostic importance. While the link between baseline LA enlargement and ablation failure is well established, prior data on post-procedural LA size changes have been limited and inconclusive. Our findings suggest that an LA that remains enlarged or fails to appreciably decrease in size after ablation is an important risk marker for arrhythmia recurrence. The incremental novelty of our work is the emphasis on standardized serial LAVI (pre-ablation and early post-ablation) as a practical marker of reverse remodeling linked to late recurrence in routine clinical follow-up.

### 4.1. Pre- and Post-Ablation LA Size as Predictors of AF Recurrence

Consistent with previous literature, a larger pre-ablation LA dimension was strongly associated with higher recurrence risk. In our cohort, baseline LAVI was approximately 9 mL/m^2^ higher in patients with late recurrence than in those without, and this separation persisted at follow-up [5]. More importantly, LA size measured at 6 and 12 months post-ablation also emerged as an independent predictor: patients with late recurrence had an average post-ablation LA size approximately 3 mm larger than those without recurrence. In separate multivariable Cox models, both pre-ablation LAVI (HR per 1 mL/m^2^ increase) and post-ablation LA diameter (HR 1.031 per mm increase) remained significant predictors along with clinical factors. These results underscore that structural remodeling of the atrium, both pre-existing and persisting after the procedure, influences long-term rhythm outcomes [9]. We identified LAVI cut-offs of 41.6 mL/m^2^ at baseline and 38.6 mL/m^2^ at 6 months that best discriminated late recurrence risk, although discrimination was modest (AUC 0.622 and 0.597). In multivariable Cox models, each 1 mL/m^2^ increase in pre-ablation LAVI was associated with a higher hazard of late recurrence (HR 1.015), and post-ablation LAVI showed a similar association (HR 1.011), suggesting that persistently increased LA size after ablation reflects limited reverse remodeling and a higher likelihood of arrhythmia recurrence.

Importantly, we also confirmed other clinical predictors of recurrence in our analysis. A history of persistent AF was associated with roughly a 1.8-fold higher risk of LR, and patients with prior typical AFL had about a 1.5-fold higher recurrence risk [3]. These factors were independent of LA size, suggesting that both the arrhythmia type and the atrial substrate are critical determinants of ablation success. Persistent AF is well known to portend higher failure rates after ablation, likely reflecting a more advanced atrial remodeling and fibrotic substrate [6]. Our data echo these observations: nearly 39% of patients with late recurrence had persistent AF, compared with ~20% of those without recurrence. Similarly, a history of AFL may indicate an underlying propensity for macro–reentrant circuits or atrial scar, which could contribute to AF recurrence despite PV isolation. By identifying these risk markers—enlarged LA (before and after ablation), persistent AF, and prior flutter—our study provides a comprehensive view of patient factors that predispose to LR. The modeling strategy was designed to separately evaluate the prognostic value of baseline and follow-up LA size while avoiding collinearity between them. This approach, together with variable inclusion based on both statistical and clinical relevance, supports the robustness of our conclusions. Diagnostic tests confirmed that the proportional hazard assumption was met, multicollinearity was minimal, and model selection based on the AIC achieved an optimal balance between model fit and parsimony. These findings support the robustness of our multivariable models and reinforce the validity of the observed associations between LA size, arrhythmia type, and recurrence risk.

### 4.2. Clinical Implications for Risk Stratification and Management

These findings carry practical implications for post-ablation patient management and follow-up. Baseline LA size is often measured as part of pre-procedure workup, and our results reinforce that an enlarged LA should heighten expectations of recurrence and encourage vigilant follow-up. More innovatively, re-assessing LA size in the months following ablation may offer additional prognostic value. In patients whose LA remains dilated (e.g., ≥39–40 mm in diameter) at the 6- and 12-month follow-up echocardiogram, the risk of later AF recurrence appears higher. This simple measurement could be incorporated into follow-up visits as an early indicator of suboptimal structural remodeling [9,10]. Patients demonstrating minimal LA reverse remodeling might warrant closer monitoring (such as more frequent ECGs/Holter checks or long-term rhythm monitoring) and aggressive risk factor modification (weight management, blood pressure control, etc.), given their elevated recurrence risk. It may also be reasonable to consider adjunctive therapy in high-risk individuals—for example, extended use of antiarrhythmic drugs or planning for a repeat ablation—if the post-ablation LA dimension remains high. Our data suggest that even after a technically successful ablation, the trajectory of LA size provides a window into the likelihood of maintaining sinus rhythm and could thus help personalize post-procedure management.

From a risk-stratification standpoint, our study underscores the importance of an integrative approach. A patient with persistent AF, prior AFL, and an enlarged LA (either initially or on follow-up) represents a profile at particularly high risk for recurrence. Identifying such individuals early can prompt tailored interventions. For instance, an AF patient with markedly enlarged LA might be counseled about the probability of needing multiple procedures or continued antiarrhythmic therapy despite ablation. Meanwhile, observing a substantial reduction in LA size after ablation (signifying favorable reverse remodeling) could reassure clinicians and patients that sinus rhythm maintenance is likely, although continued surveillance is still required. LA size before and after ablation—alongside key clinical factors—can be used to refine risk stratification after AF ablation, enabling proactive management adjustments to improve long-term outcomes.

### 4.3. Multimodality Assessment of LA Volume and Intraprocedural Mapping

Beyond transthoracic echocardiography, LA size can be quantified using cross-sectional imaging (computed tomography or cardiac magnetic resonance) and can also be estimated intraprocedurally from three-dimensional electroanatomic mapping geometries. Zero-fluoroscopy strategies that combine 3D mapping with intracardiac echocardiography have become increasingly feasible in contemporary practice [8]. However, electroanatomic “intracavitary” LA volumes are influenced by map acquisition (including point density and segmentation) and typically yield larger values than echocardiographic volumes; therefore, values obtained by mapping and echocardiography should not be considered interchangeable when interpreting LA remodeling over time [8]. In this study we focused on serial echocardiographic LAVI because it is widely available and standardized for follow-up. Future prospective studies that collect and compare electroanatomic mapping-derived volumes with echocardiography and CMR may clarify whether intraprocedural chamber volume adds incremental prognostic information beyond LAVI alone.

### 4.4. Comparison with Previous Studies on AF Recurrence Following LA Ablation

Our results both corroborate and extend prior findings on predictors of AF ablation outcomes. The association of baseline LA enlargement with recurrence has been consistently reported in the literature. A meta-analysis by Njoku et al. found that patients with AF recurrence had, on average, significantly larger LA volumes (~7 mL greater) than those who remained in sinus rhythm [5]. Similarly, many individual studies have identified LA diameter or volume as a strong predictor of ablation failure, reflecting the concept that a structurally remodeled atrium provides fertile ground for AF persistence. Our observed recurrence rate of ~38.5% at 1 year (after a single ablation) is in line with previously reported success rates; typically, about 60–70% of patients maintain sinus rhythm after one ablation (around 59% in paroxysmal AF, lower in persistent AF), with the remainder experiencing recurrence [11]. This context highlights that our cohort’s outcomes are representative of contemporary practice and that our identified predictors address a common clinical challenge.

Data on post-ablation LA size and remodeling have emerged only recently. Some studies have shown that successful AF ablation is associated with reduced LA volume and improved function, particularly in patients with persistent AF. For example, a 2022 study by Liu et al. reported that, in persistent and long-standing persistent AF, LA diameter and volume significantly decreased within one year after ablation (evidence of reverse remodeling), whereas paroxysmal AF patients showed no significant structural change [6]. Our finding that LA size at follow-up carries prognostic weight aligns with the notion that the degree of reverse remodeling post-ablation is meaningful. In fact, a recent subanalysis from the DECAAF II trial (in persistent AF patients) demonstrated that the mean LA volume index decreased by approximately 10 mL/m^2^ after ablation, and importantly, the magnitude of LA volume reduction correlated with better outcomes—patients with greater LA shrinkage had lower arrhythmia recurrence and reduced AF burden on follow-up [9]. This reinforces our result that a smaller (or shrinking) atrium post-ablation is a favorable sign. However, the literature has not been unanimous: other analyses have found inconsistent relationships between changes in LA size and long-term recurrence, suggesting that multiple factors (extent of fibrosis, arrhythmia type, etc.) modulate this relationship [12]. Our study adds to this evolving literature by providing evidence from a sizable cohort that both baseline and follow-up LA metrics are relevant, and it underscores the value of monitoring structural remodeling as part of post-ablation evaluation.

The higher recurrence rates in persistent AF patients are well documented in clinical trials and registries, and our data echo these observations (nearly 39% of patients with LR had persistent AF vs. approximately 20% without recurrence). Prior studies have explained this by the greater atrial fibrosis and electrical remodeling in persistent AF, which make achieving durable PVI and non-inducibility more challenging. Similarly, although the prognostic impact of prior typical AFL has been less emphasized, our findings highlight it as an important marker of atrial arrhythmic propensity [13]. Some investigators have shown that even after successful cavotricuspid isthmus ablation for flutter, many patients go on to manifest AF, implying common underlying substrates [14]. In the context of AF ablation, a patient with prior flutter might have a substrate prone to developing atypical flutters or gaps in ablation lines, contributing to arrhythmia recurrence. Thus, our results are consistent with the broader literature while shedding light on the underexplored factor of post-ablation LA size, thereby expanding the discussion on how to predict and improve long-term ablation success.

### 4.5. Potential Mechanisms of LA Remodeling and Recurrence

The link between LA size and AF recurrence likely reflects underlying pathophysiologic mechanisms of atrial myopathy and fibrosis. AF causes structural remodeling of the atria, characterized by myocyte loss, fibrotic tissue deposition, and chamber dilatation [11]. LA enlargement is therefore both a marker and a contributor of this atrial myopathy, creating a substrate that sustains arrhythmias. Patients with larger atria pre-ablation are presumed to harbor more extensive fibrotic remodeling; this can manifest as areas of low voltage and conduction slowing that are not fully eliminated by standard ablation lesions, providing a nidus for AF to reinitiate. Indeed, LA enlargement has been called an important marker of atrial fibrosis and myopathy [9]. When sinus rhythm is maintained after ablation, some degree of reverse remodeling is expected—the reduction in stretch and tachyarrhythmia-related injury allows the atrium to partially recover, which can translate into a smaller LA size and improved mechanical function. Our findings support this concept: among patients who remained free of AF, follow-up LA dimensions tended to be lower (and LVEF slightly higher) compared to those who recurred, suggesting beneficial remodeling in the non-recurrence group. Conversely, in patients with recurrence, the continued episodes of AF (even if intermittent) or ongoing atrial pathology may prevent substantial reverse remodeling; their LA size remained enlarged on follow-up, and even left ventricular function failed to improve (and was in fact lower than in the non-recurrence group). This disparity in post-ablation cardiac remodeling between the two groups highlights that recurrence is not merely an electrical failure but is accompanied by persistence of adverse structural and functional changes.

Atrial fibrosis plays a central role in these observations. Patients with recurrent arrhythmia likely had a higher fibrotic burden that was not resolved by ablation, whereas those without recurrence had more effective modification of the arrhythmogenic substrate or inherently less fibrosis [15]. Persistent AF, as an independent predictor, aligns with this reasoning: by definition, longer-standing persistent AF causes more fibrosis and scar within the atrium, which can be pro-arrhythmic even after acute PVI. Similarly, a history of AFL might indicate a pre-existing scar (e.g., in the right atrium or LA) that signifies a propensity for reentrant arrhythmias. These substrate differences may explain why a history of persistent AF and flutter contributed to the recurrence risk, alongside LA size, in our study. The interplay between ablation lesions and structural remodeling is another mechanistic consideration. Successful ablation itself can cause intentional scarring that electrically isolates triggers and may even reduce chamber size. Some evidence suggests that the decrease in LA volume observed after ablation is not solely due to reverse remodeling from maintained sinus rhythm, but also partly due to the direct effect of ablation-induced scar contracting the atrial walls [10,16,17]. In other words, ablative injury can structurally “tighten” the atrium. In patients who undergo extensive lesion sets (such as those with substrate modification for persistent AF), greater LA downsizing may occur due to scar formation. In our cohort, LA size changes varied considerably between groups. This divergence suggests that those who maintained sinus rhythm likely benefited from both reverse remodeling (from relief of AF-related atrial stretch) and ablation-related scar contraction, while those with recurrence experienced limited remodeling and may have developed further structural pathology. The resulting lack of LA size reduction in the recurrence group reinforces its prognostic value. Taken together, these pathophysiologic insights suggest that LA size is a surrogate for the burden of atrial disease: a smaller post-ablation LA likely reflects effective rhythm control and healthier atrial myocardium, whereas a larger LA (pre- or post-ablation) indicates substantial atrial myopathy that predisposes individuals to AF’s return.

### 4.6. Potential Impact of CAD on AF Recurrence

Although our study did not specifically evaluate CAD as a predictor of AF recurrence, recent evidence suggests that CAD contributes to AF maintenance through ischemia-driven atrial remodeling and fibrosis. Notably, independent analyses have shown that markers of significant coronary atherosclerosis, such as elevated coronary artery calcium scores, are associated with a substantially increased risk of AF recurrence following catheter ablation [18]. Given the frequent coexistence of these conditions, recognizing CAD as a potential factor in AF outcomes is crucial, even as the exact causal relationship continues to be refined in recent literature [19].

### 4.7. Secondary AF Precipitants and Potential Confounding

AF may arise in the setting of acute “secondary” precipitants (e.g., pneumonia/sepsis, respiratory failure, pneumothorax, myocarditis, pericarditis, alcohol intoxication, thyrotoxicosis, cardiothoracic surgery, or other surgery), and these episodes may have distinct mechanisms and downstream management compared with primary AF [20]. Importantly, such transient inflammatory or metabolic triggers could influence arrhythmia recurrence risk independently of atrial size and may also affect the interpretation of post-ablation remodeling. Because our retrospective dataset did not systematically adjudicate secondary precipitants at index presentation or during follow-up, residual confounding related to secondary AF triggers cannot be fully excluded. Prospective studies incorporating standardized capture of secondary precipitants and inflammatory/metabolic markers will be helpful to further refine the independent prognostic role of serial LAVI.

### 4.8. Limitations

Several limitations of our study should be acknowledged. First, this was a retrospective, single-center study, which inherently carries risks of selection bias and limits generalizability. The patient population and ablation protocols employed at our institution reflect our practice and may differ from those at other centers. Moreover, our patient inclusion spanned 2013 to 2021—a period during which AF ablation techniques advanced—potentially limiting the applicability of outcomes from earlier cases to contemporary practice. A related limitation is the moderate sample size (408 patients); while sufficient to detect significant predictors, the cohort may not capture less common predictors or allow extensive subgroup analyses. Second, advanced imaging such as late gadolinium enhancement cardiac magnetic resonance imaging was not performed, so we could not directly quantify atrial fibrosis or scar—factors that likely play a crucial role in recurrence but were unmeasured in our study. Incorporating such data might improve risk stratification beyond what LA diameter alone can offer. Third, follow-up echocardiography was performed at 6 and 12 months post-ablation; however, we did not capture the dynamic trajectory of LA size changes beyond that window. Some patients may exhibit delayed remodeling or dilation after the 12-month mark (for example, if AF recurs late). Fourth, our monitoring for AF recurrence, while consistent with guideline practice (scheduled clinic ECGs and 24-h Holter monitoring were performed at 3 to 6 months post-ablation and additionally in patients presenting with arrhythmic symptoms), may have missed asymptomatic or paroxysmal episodes, potentially underestimating recurrence and biasing classification. This means some “non-recurrence” patients might have had undocumented AF between visits. More intensive rhythm monitoring (implantable loop recorders or frequent prolonged external monitoring) could provide a more granular measure of AF burden and might strengthen associations with structural findings. Another limitation is that we did not systematically evaluate procedural variables such as the lesion set, ablation extent, or achieved lesion durability (apart from acute PVI), nor did we collect detailed data on post-ablation medication use; thus, the influence of medications on AF recurrence could not be evaluated. In addition, procedural strategies were individualized (e.g., posterior wall isolation and substrate modification in selected patients), which may have influenced both LA remodeling and recurrence risk. We also did not capture contact force–related parameters, which may influence lesion durability and pulmonary vein isolation success. Furthermore, because all patients underwent radiofrequency ablation, our findings may not be directly generalizable to alternative energy sources such as cryoballoon or pulsed field ablation. Additionally, we did not specifically analyze the influence of underlying coronary artery disease on AF recurrence; however, the prevalence of CAD was low and similar between patients with and without recurrence in our cohort. Fifth, as an observational study, this study could only demonstrate associations, not causation. For example, while we found that less post-ablation LA remodeling is linked to recurrence, it remains uncertain whether interventions that promote LA reverse remodeling would causally reduce recurrence, or whether LA size is merely a bystander marker of other pathologic processes. These limitations highlight the need to interpret our findings with appropriate caution. As left atrial strain may reflect functional remodeling beyond structural enlargement, future studies incorporating strain parameters are warranted. Finally, although both pre- and post-ablation LA size were significant predictors of LR, their discriminative power was modest, indicating that risk stratification should be multifactorial. We recognize that LA volume is a more comprehensive measure of atrial size and can be a better predictor in some contexts. Moreover, given the retrospective single-center design and the lack of direct assessment of atrial scar or fibrosis, prospective multi-center studies are warranted. Future investigations integrating advanced imaging (e.g., late gadolinium enhancement magnetic resonance imaging) and atrial functional indices may further clarify the prognostic role of structural remodeling and guide tailored interventions to improve outcomes in patients with enlarged atria [21]. These findings support the incorporation of post-ablation echocardiographic follow-up as a practical tool for early risk stratification. Thus, early post-ablation LA size assessment can help flag high-risk patients while there is still an opportunity for intervention.

## 5. Conclusions

In conclusion, both baseline and post-ablation LA size independently predict late AF recurrence. Larger or persistently dilated LA indicates greater recurrence risk, whereas a reduction in LA size reflects favorable remodeling. Recognizing the dynamic relationship between LA structure and rhythm outcome may help refine personalized strategies to enhance long-term sinus rhythm maintenance after ablation.

## Figures and Tables

**Table 1:** Baseline Characteristics and Echocardiographic Parameters in Patients With and Without Late Recurrence.

Variables	With LR (*n* = 157)	Without LR (*n* = 251)	*p* Value
Age (yrs)	60.7 ± 9.8	59.9 ± 10.8	0.823
Male gender	56.1% (88)	64.1% (161)	0.103
Atrial flutter	36.9% (58)	21.5% (54)	<0.001
Persistent AF	38.9% (61)	19.5% (49)	<0.001
Hypertension	54.1% (85)	50.6% (127)	0.541
Diabetes mellitus	24.2% (38)	17.5% (44)	0.102
Heart failure	13.4% (21)	8.8% (22)	0.140
Old stroke	12.7% (20)	7.6% (19)	0.084
Coronary artery disease	12.1% (19)	11.2% (28)	0.771
CHA2DS2-VASc score	2.2 ± 1.5	1.8 ± 1.5	0.019
Echocardiographic data			
LAVI (mL/m^2^) before RFA	46.1 ± 22.3	36.9 ± 15.6	<0.001
LAVI (mL/m^2^) after RFA	44.6 ± 25.6	36.2 ± 17.5	<0.001
LVEF (%) before RFA	64.8 ± 12.0	67.8 ± 9.9	0.074
LVEF (%) after RFA	65.6 ± 11.4	69.7 ± 8.8	0.008

The data are presented as means ± standard deviations or numbers (percentages). AF = atrial fibrillation, LAVI = left atrial volume index, LR = late recurrence, LVEF = left ventricular ejection fraction, RFA = radiofrequency ablation.

**Table 2:** Multivariable Cox proportional hazards models for late recurrence.

Variables	HRs	95% CI	*p* Value
Model 1			
AFL	1.475	1.062–2.049	0.021
Persistent AF	2.046	1.439–2.909	<0.001
LAVI before RFA	1.014	1.007–1.022	<0.001
Model 2			
AFL	1.555	1.091–2.215	0.015
Persistent AF	1.810	1.264–2.593	0.001
LAVI after RFA	1.010	1.003–1.018	0.007

Multivariable Cox proportional hazards regression was conducted to assess associations between recurrence and clinical factors. Results are presented as hazard ratios with 95% confidence intervals and corresponding *p* values. AF = atrial fibrillation, CI= confidence interval, HRs = hazard ratios, LA = left atrial, LAVI = left atrial volume index, LVEF = left ventricular ejection fraction, RFA = radiofrequency ablation Model 1: Baseline characteristics and pre-RFA echo data with *p* < 0.1, including AFL, PerAF, Stroke, CHA2DS2VASc score ≥ 2, Pre-RFA LA size, LVEF Model 2: Baseline characteristics and post-RFA echo data with *p* < 0.1, including AFL, PerAF, Stroke, CHA2DS2VASc score ≥ 2, Post-RFA LA size, LVEF.

## Data Availability

The data supporting the findings of this study are available from the corresponding author upon reasonable request.

## Supplementary Materials

The following supporting information can be downloaded at: https://www.mdpi.com/article/10.3390/diagnostics16040628/s1, Figure S1: Schoenfeld residuals for assessing the proportional hazards assumption; Table S1: Stepwise selection guided by the Akaike Information Criterion (AIC) for model optimization; Table S2: Assessment of multicollinearity using variance inflation factors (VIF).

## Abbreviations

AF = atrial fibrillation, HF = heart failure, LA = left atrium, RFA = radiofrequency catheter ablation, PV = pulmonary vein, PVI = pulmonary vein isolation, AT = atrial tachycardia, AFL = atrial flutter, ECG = electrocardiogram, LVEF = left ventricular ejection fraction, ER = early recurrence, LR = late recurrence, ROC = receiver operating characteristic, AUC = area under the curve, CI = confidence interval, HR = hazard ratio, AIC = Akaike Information Criterion, VIF = variance inflation factors.
