# Supplementary material for: Left Atrial Size Modification After Catheter Ablation Predicts Late Atrial Fibrillation Recurrence
Source: Diagnostics (Basel). 2026 Feb 21;16(4):628. doi: 10.3390/diagnostics16040628 (PMC12939483; doi:10.3390/diagnostics16040628)
Supplement: Supplementary file 1 [file diagnostics-16-00628-s001.zip › diagnostics-4131155-supplementary.pdf]

Supplementary Figure S1. Schoenfeld residuals for assessing the proportional hazards assumption.

The global test yields p-values > 0.05 across covariates, indicating no statistically significant deviation from proportionality. This supports the validity of the proportional hazards assumption in the Cox regression model.

Model 1:

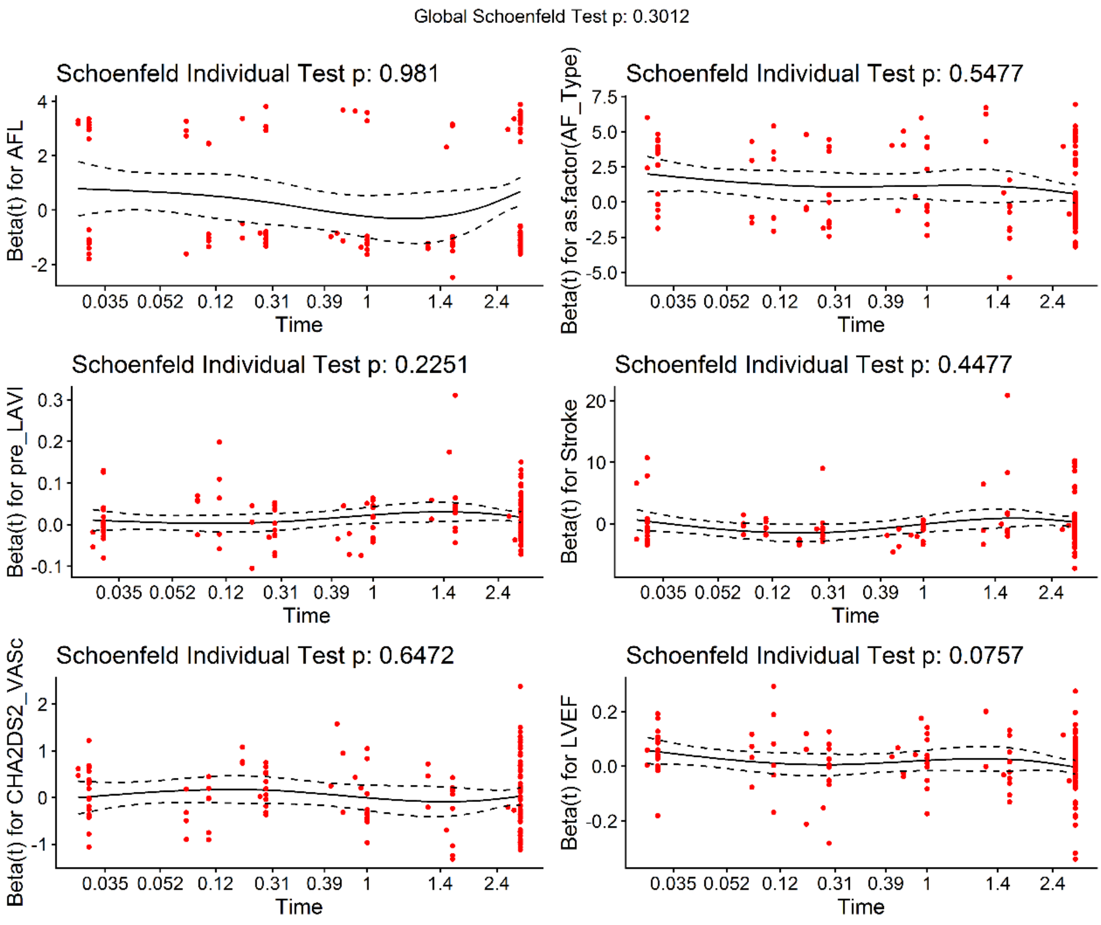

Model 2:

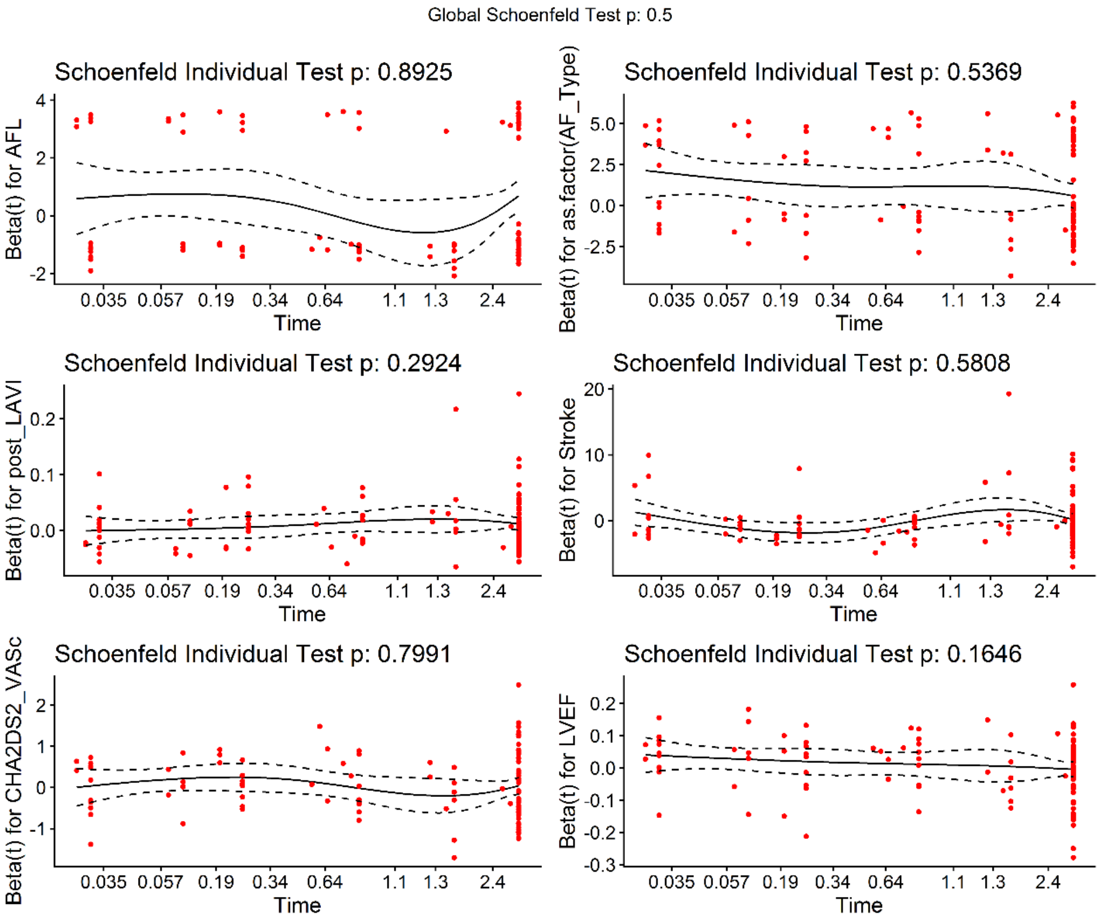

Global Schoenfeld Test p: 0.5
Schoenfeld Individual Test p: 0.8925
Beta(t) for AFL
Time
Schoenfeld Individual Test p: 0.5369
Beta(t) for as.factor(AF_Type)
Time
Schoenfeld Individual Test p: 0.2924
Beta(t) for post_LAVI
Time
Schoenfeld Individual Test p: 0.5808
Beta(t) for Stroke
Time
Schoenfeld Individual Test p: 0.7991
Beta(t) for CHA2DS2_VASc
Time
Schoenfeld Individual Test p: 0.1646
Beta(t) for LVEF
Time
0.035 0.057 0.19 0.34 0.64 1.1 1.3 2.4

Supplementary Table S1. Stepwise selection guided by the Akaike Information Criterion (AIC) for model optimization.

Sequential AIC-based selection identified the final set of covariates that best balanced model fit and parsimony. Variables retained in the final model demonstrated optimal contribution to predictive performance while minimizing overfitting.

Model 1: AIC=1521.27

	coef	exp(coef)	se(coef)	z	Pr(>|z|)
AFL	0.466907	1.595053	0.177383	2.632	0.008483
as.factor(AF_Type)1	0.608471	1.837619	0.210553	2.890	0.003854
as.factor(AF_Type)2	0.945562	2.574260	0.245293	3.855	0.000116
Pre_LAVI	0.015035	1.015148	0.004315	3.484	0.000494

	exp(coef)	exp(-coef)	lower0.95	upper0.95
AFL	1.595	0.6269	1.127	2.258
as.factor(AF_Type)1	1.838	0.5442	1.216	2.776
as.factor(AF_Type)2	2.574	0.3885	1.592	4.163
Pre_LAVI	1.015	0.9851	1.007	1.024

Model 2: AIC=1229.37

	coef	exp(coef)	se(coef)	z	Pr(>|z|)
AFL	0.467815	1.596502	0.194283	2.408	0.01604
as.factor(AF_Type)1	0.705071	2.023990	0.217896	3.236	0.00121
as.factor(AF_Type)2	0.743139	2.102525	0.281685	2.638	0.00833
Post_LAVI	0.010813	1.010872	0.003827	2.826	0.00472

	exp(coef)	exp(-coef)	lower0.95	upper0.95
AFL	1.597	0.6264	1.091	2.336
as.factor(AF_Type)1	2.024	0.4941	1.320	3.102
as.factor(AF_Type)2	2.103	0.4756	1.211	3.652
Post_LAVI	1.011	0.9892	1.003	1.018

Supplementary Table S2. Assessment of multicollinearity using variance inflation factors (VIF).

All included variables demonstrated acceptable levels of collinearity, with VIF values below 2.0, indicating no evidence of significant multicollinearity in the final model.

Model 1:

	GVIF	Df	GVIF^(1/(2*Df))
AFL	1.024252	1	1.012053
as.factor(AF_Type)	1.268487	2	1.061259
Pre_LAVI	1.18954	1	1.09066
Stroke	1.391355	1	1.179557
CHA2DS2_VASc	1.40986	1	1.187375
LVEF	1.227157	1	1.107771

Model 2:

	GVIF	Df	GVIF^(1/(2*Df))
AFL	1.017976	1	1.008948
as.factor(AF_Type)	1.228011	2	1.05269
Post_LAVI	1.193297	1	1.092382
Stroke	1.513994	1	1.230445
CHA2DS2_VASc	1.623504	1	1.274168
LVEF	1.158778	1	1.076465
